# Primary Renal Ewing Sarcoma Presenting With Divergent Radiological Phenotypes: A Radiology-Focused Case Series Featuring the Mimicking of Renal Cell Carcinoma and Urinary Tract Urothelial Carcinoma

**DOI:** 10.7759/cureus.114625

**Published:** 2026-08-16

**Authors:** Ketki Kedar, Deepshikha Arora, Avinash Dhok, Saikat Mitra, Nisha B Meshramm

**Affiliations:** 1 Department of Radiodiagnosis and Interventional Radiology, All India Institute of Medical Sciences, Nagpur, IND; 2 Department of Pathology, All India Institute of Medical Sciences, Nagpur, IND

**Keywords:** ewing sarcoma (es), ewing sarcoma family of tumors(esft), extraosseous ewing's sarcoma, genito urinary cancer, primitive neuroectodermal tumor (pnet), renal cancer, renal cell carcinoma (rcc), renal ewing sarcoma, transitional cell carinoma

## Abstract

Primary renal Ewing sarcoma/primitive neuroectodermal tumor (PNET) is an exceptionally rare and highly aggressive malignancy belonging to the Ewing sarcoma family of tumors (ESFT). Owing to its nonspecific clinical and imaging appearance, it frequently mimics more common renal malignancies, particularly renal cell carcinoma (RCC), resulting in diagnostic difficulty and delayed therapeutic decision-making.

We present a radiology-oriented case report of two female patients with histopathologically confirmed primary renal Ewing sarcoma demonstrating markedly different imaging phenotypes. The first patient presented with a large infiltrative renal mass showing extensive venous tumor thrombus closely simulating aggressive RCC. The second patient, presenting in the elderly age group, demonstrated diffuse pelvicalyceal and ureteric involvement with preservation of renal contour and contiguous urothelial extension, closely resembling upper tract urothelial carcinoma/transitional cell carcinoma (TCC).

Both tumors demonstrated large size, heterogeneous enhancement, internal necrosis, an infiltrative growth pattern, and aggressive locoregional disease. Positron emission tomography-computed tomography (PET-CT) demonstrated intense fluorodeoxyglucose (FDG) avidity and metastatic disease in both cases. Histopathological and immunohistochemical (IHC) analysis confirmed Ewing sarcoma/PNET in both patients.

This case series highlights the broad radiologic spectrum of primary renal Ewing sarcoma and emphasizes imaging features that should raise suspicion for this rare diagnosis, including large rapidly enlarging infiltrative renal masses, disproportionate necrosis, marked vascular involvement, diffusion restriction on MRI, early metastatic disease, and atypical imaging behavior not entirely conforming to conventional RCC or urothelial malignancy. Awareness of these features may facilitate earlier diagnosis and appropriate multidisciplinary management.

## Introduction

The Ewing sarcoma family of tumors (ESFT) comprises a spectrum of highly aggressive malignant small round cell neoplasms, including Ewing sarcoma of bone, extraosseous Ewing sarcoma, Askin tumors, and primitive neuroectodermal tumor (PNET) [1,2]. These tumors share common histopathological, immunohistochemical (IHC), and cytogenetic characteristics, most notably translocations involving the Ewing sarcoma breakpoint region 1 (EWSR1) gene, particularly t(11;22)(q24;q12), resulting in EWSR1-FLI1 fusion [2].

While the majority of ESFTs arise from osseous structures in children and young adults, extraskeletal Ewing sarcoma (EES) constitutes a rare subgroup, accounting for approximately 15% to 20% of all ESFTs [2]. Renal involvement is exceedingly uncommon, with fewer than 120-150 cases reported in the literature to date [1,3-5]. Primary renal Ewing sarcoma typically affects adolescents and young adults and demonstrates an aggressive clinical course characterized by rapid growth, early metastasis, vascular invasion, and poor overall prognosis [1,4-7].

Clinical presentation is nonspecific and commonly includes abdominal pain, flank pain, palpable abdominal mass, hematuria, constitutional symptoms, or incidentally detected renal masses [1,5-8]. Imaging findings are similarly nonspecific and frequently overlap with more common renal malignancies such as renal cell carcinoma (RCC), Wilms tumor, renal lymphoma, sarcomas, and urothelial carcinoma [3,5,8]. Consequently, preoperative radiologic diagnosis remains challenging in most cases.

Radiologically, renal Ewing’s sarcoma commonly presents as a large heterogeneous renal mass with necrosis, hemorrhage, an infiltrative growth pattern, and varying degrees of vascular invasion [2,3,6,7]. The MRI frequently demonstrates marked diffusion restriction owing to the hypercellular nature of the tumor [3]. Fluorodeoxyglucose (FDG) PET-CT is useful for staging and evaluation of metastatic disease [2].

We present two histopathologically confirmed cases of primary renal Ewing sarcoma demonstrating divergent radiologic phenotypes. One case closely mimicked aggressive RCC with extensive venous tumor thrombosis, while the other case simulated urothelial carcinoma with diffuse pelvicalyceal and ureteric involvement in an elderly patient. Through this case series, we aim to highlight the broad radiologic spectrum of renal Ewing sarcoma and discuss imaging clues that may aid radiologists in suspecting this rare diagnosis.

## Case presentation

Case one

A 42-year-old female presented with progressively worsening dull abdominal pain for six months. The pain was localized to the right lumbar region, radiating to the right iliac fossa. Over the preceding month, she had also noticed a gradually enlarging abdominal lump. There was no history of hematuria, fever, lithuria, or lower urinary tract symptoms. Her past medical history was significant for hypertension on regular medical therapy and a cesarean section eleven years earlier. Laboratory investigations revealed mild anemia with a hemoglobin level of 9.9 g/dL. Renal function tests were within normal limits, with serum creatinine measuring 0.76 mg/dL.

**Figure 1:**
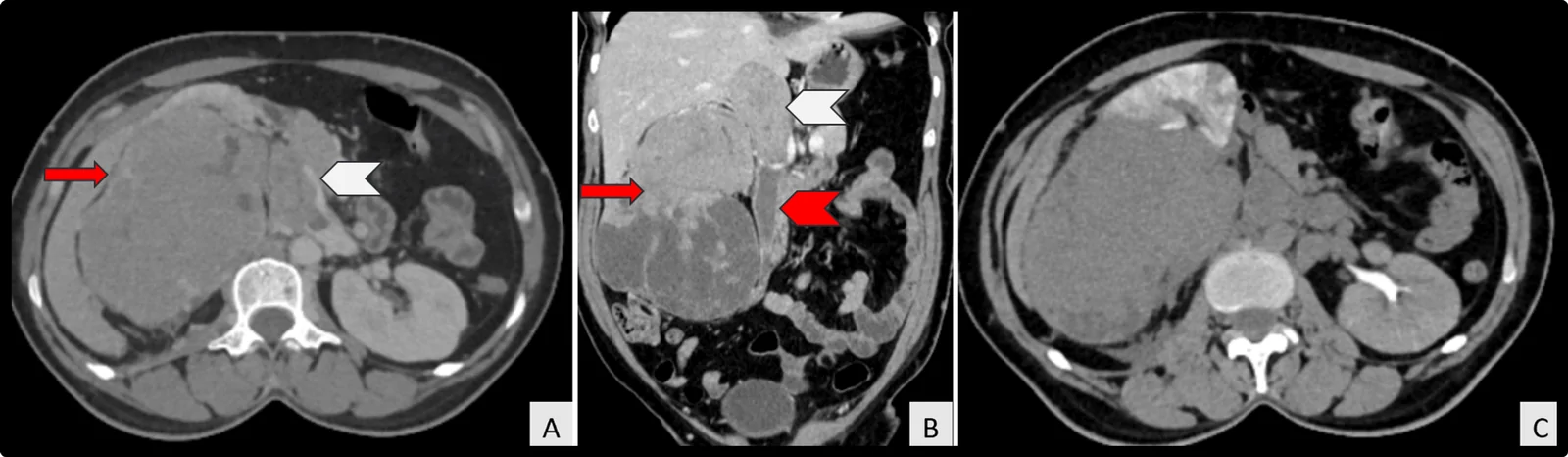
CECT urography demonstrating the primary renal mass and venous involvement A: Axial nephrographic phase of CT urography image demonstrates a large heterogeneously enhancing right renal mass (red arrow) with contiguous tumor thrombus extending into the right renal vein and inferior vena cava (IVC) (white arrowhead). B: Coronal reconstructed CT urography image showing mass lesion (red arrow) and extension of the enhancing thrombus from the right renal vein into the IVC (white arrowhead), as well as non-enhancing bland thrombus in the rest of the IVC (red arrowhead). C: Delayed excretory-phase CT urography image demonstrates absence of contrast excretion from the right kidney, consistent with a non-functioning obstructed kidney secondary to extensive tumor involvement. CECT: Contrast-enhanced computed tomography

**Figure 2:**
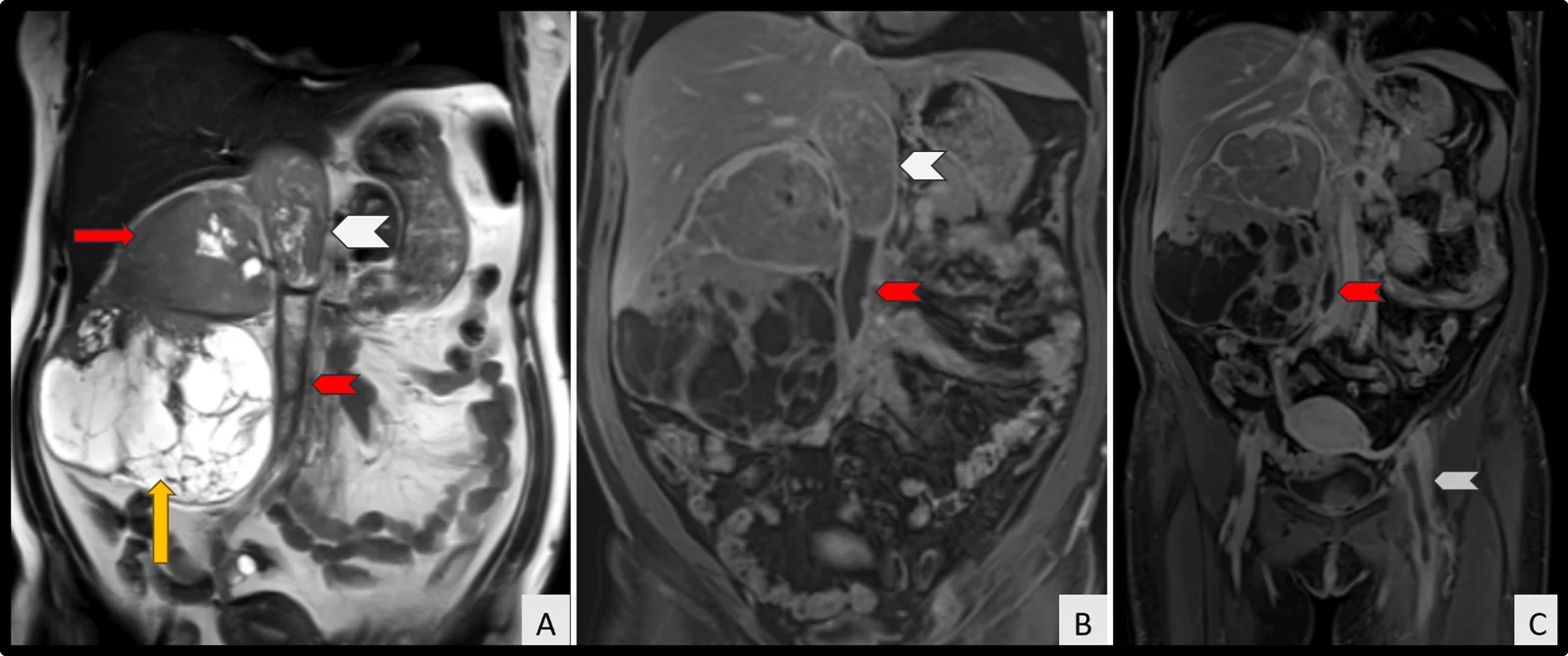
MRI of the primary renal tumor in the right kidney with venous extension A: Coronal reconstructed T2-weighted half-Fourier acquisition single-shot turbo spin echo (HASTE) image demonstrates a large heterogeneous right renal mass (red arrow) occupying the interpolar and upper polar region, with contiguous tumor thrombus extending into the right renal vein and IVC (white arrowhead). Distal to the enhancing tumor thrombus, a bland thrombus (red arrowhead) is identified within the infrahepatic IVC. The yellow arrow points to the large exophytic component of the tumor with a major necrotic component. B: Coronal reconstructed contrast-enhanced T1-weighted image demonstrates heterogeneous enhancement of the primary renal mass and contiguous enhancing tumor thrombus within the IVC (white arrowhead), while the distal infrahepatic bland thrombus remains non-enhancing(red arrowhead), confirming both types of venous thrombosis. C: Coronal reconstructed contrast-enhanced T1-weighted image demonstrates extension of venous thrombosis into the left lower limb deep venous system (green arrowhead), consistent with associated deep venous thrombosis (DVT). IVC: Inferior vena cava

Contrast-enhanced computed tomography (CECT) with CT urography protocol and contrast-enhanced MRI (CE-MRI) of the abdomen demonstrated a large, well-defined, heterogeneously enhancing lobulated soft tissue mass arising from the interpolar region of the right kidney with extension into the upper pole. The lesion demonstrated predominantly solid morphology with multiple internal non-enhancing necrotic areas. The mass showed an expansile 'ball-type' growth pattern with involvement of the renal medulla and calyceal system, along with a large exophytic component extending posteroinferiorly up to the level of the aortic bifurcation. The lesion did not show hyperenhancement on the arterial phase (Figures 1-2).

The lesion crossed the renal polar lines and demonstrated no evidence of macroscopic fat or intratumoral aneurysm formation. Few internal calcific foci were identified. Associated perinephric fat stranding and obliteration of the fat plane with the right psoas muscle suggested local infiltration.

A striking imaging feature was extensive venous involvement. Tumor extension into the renal pelvis, right renal vein, and inferior vena cava (IVC) was identified, extending superiorly up to the intrahepatic IVC segment. The distal infrahepatic IVC demonstrated an associated non-enhancing bland thrombus. In addition, bilateral gonadal veins were dilated with multiple venous collateral channels, suggesting chronic venous obstruction (Figures 1-2). Doppler ultrasonography further revealed deep venous thrombosis (DVT) involving the right lower limb (Figures 1-2). Based on imaging findings, the initial radiologic impression favored aggressive RCC with venous tumor thrombus. However, the large tumor size, marked necrosis, infiltrative morphology, extensive vascular involvement, and absence of typical hyper-vascular clear cell RCC characteristics raised suspicion for an aggressive non-epithelial neoplasm, including renal sarcoma.

**Figure 3:**
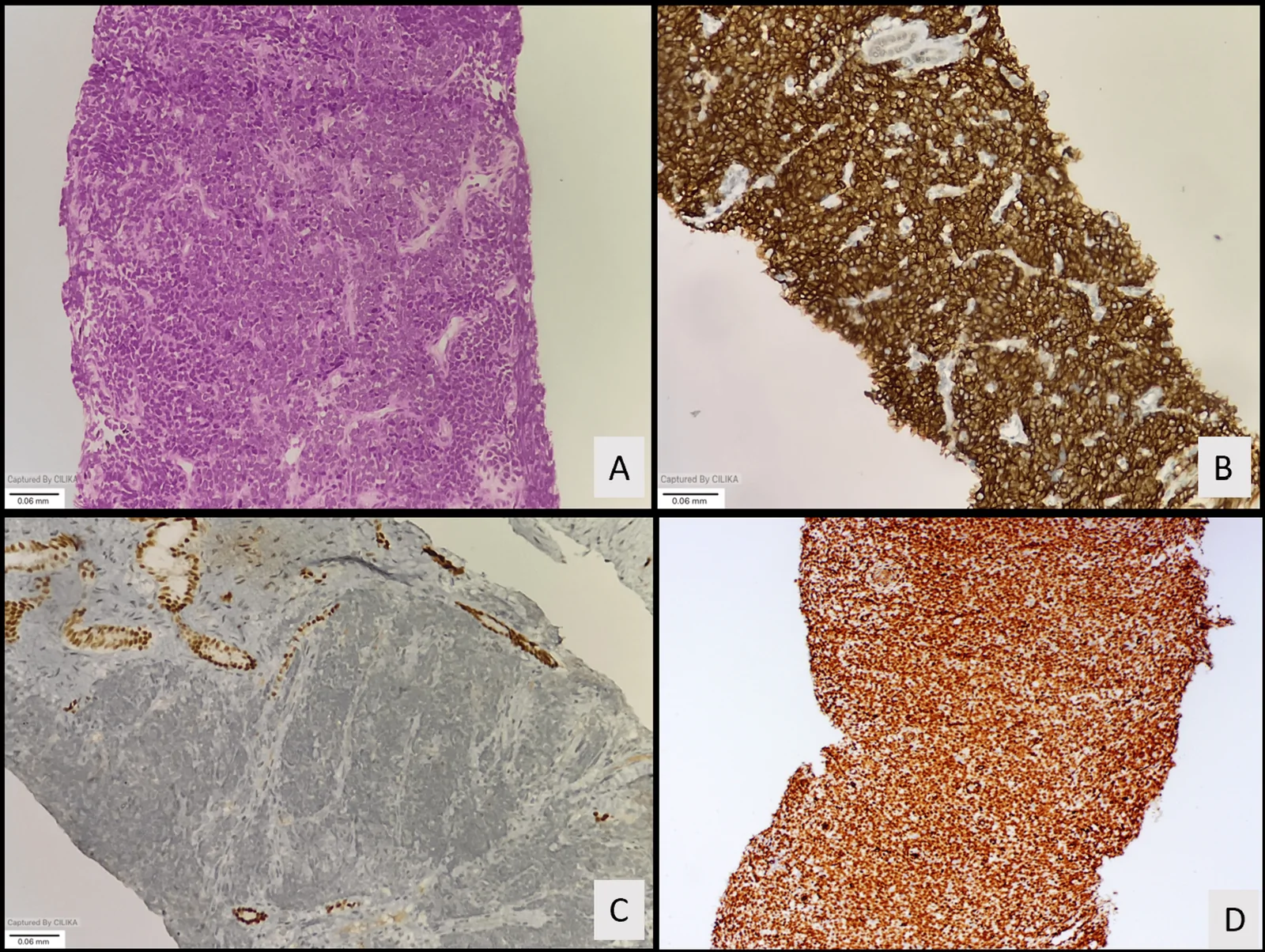
Histopathological and immunohistochemical images of primary renal Ewing sarcoma/PNET A: A sheet of small round cells with round hyperchromatic nuclei, inconspicuous nucleoli, and scant cytoplasm (HE, 100x); B: Tumor cells show diffuse, strong membranous positivity for CD99 (IHC, 100x); C: The tumor cells are negative for PAX-8, with positive internal control in the renal tubules (IHC, 100x); D: The tumor cells show strong, diffuse nuclear positivity for NKX 2.2 (IHC, 100x) PNET: Primitive neuroectodermal tumor, IHC: Immunohistochemistry

Histopathological examination of the percutaneous biopsy specimen revealed a malignant small round blue cell tumor. Immunohistochemical analysis confirmed Ewing sarcoma/PNET. The tumor cells showed strong and diffuse complete membranous positivity for CD99. Additionally, the absence of PAX8, CD45, desmin, TLE-1, S-100, CD56, and WT-1 ruled out the possibility of primary renal undifferentiated carcinoma, lymphoma, rhabdomyosarcoma, synovial sarcoma, melanoma, small cell neuroendocrine carcinoma, blastemal predominant Wilms tumor, and desmoplastic small round cell tumor. Further molecular confirmation could not be performed due to financial constraints and unavailability of fluorescence in situ hybridization (FISH)/next-generation sequencing (NGS) facilities at our center. However, NKX2.2, which is a downstream target of the EWSR1::FLI1 gene fusion associated with Ewing sarcoma, showed diffuse positivity in the tumor nuclei, confirming Ewing sarcoma (Figure 3).

**Figure 4:**
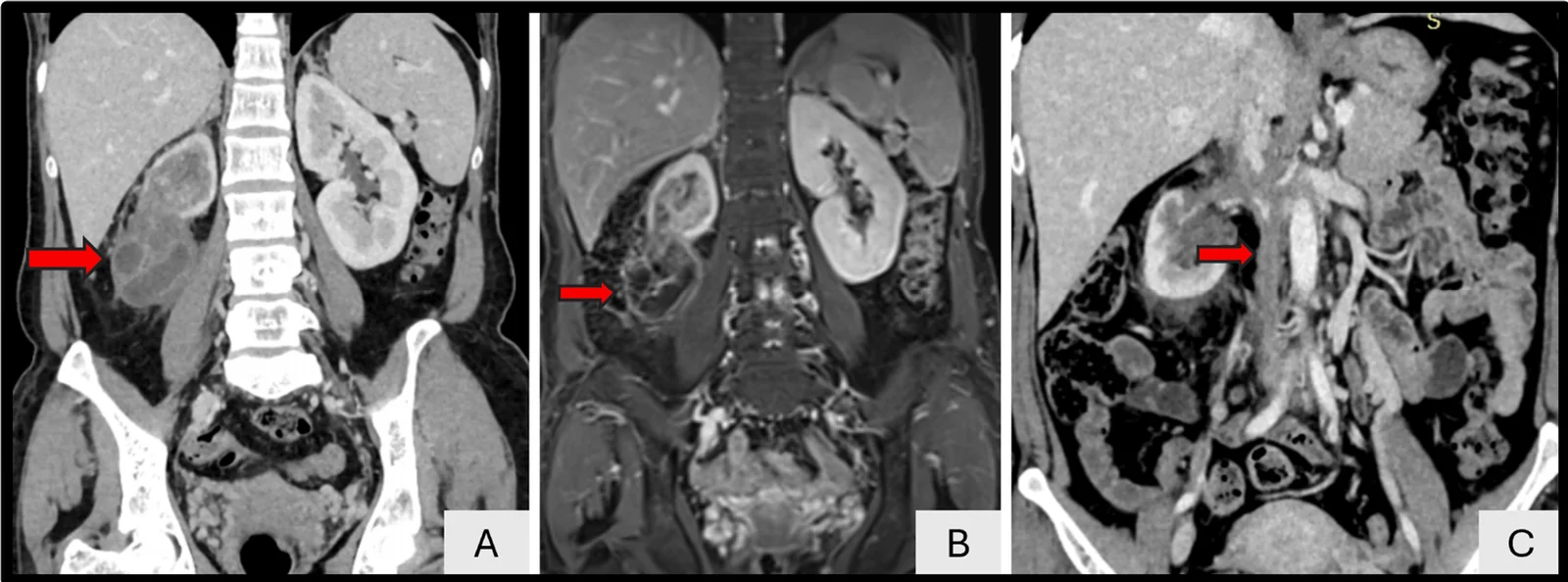
Post-treatment imaging demonstrating excellent response to systemic chemotherapy A: Coronal reconstructed cortico-medullary phase of CT urography image shows marked interval reduction in the size of the primary lesion following systemic chemotherapy with a cyclophosphamide and adriamycin-based regimen (red arrow). B: Coronal reconstructed post-contrast fat-suppressed T1-weighted MRI demonstrates significant regression of the lesion with an increase in cystic and necrotic components (red arrow). C: Coronal reconstructed corticomedullary CT urography image shows a consistent IVC tumor thrombus (red arrow) IVC: Inferior vena cava

**Figure 5:**
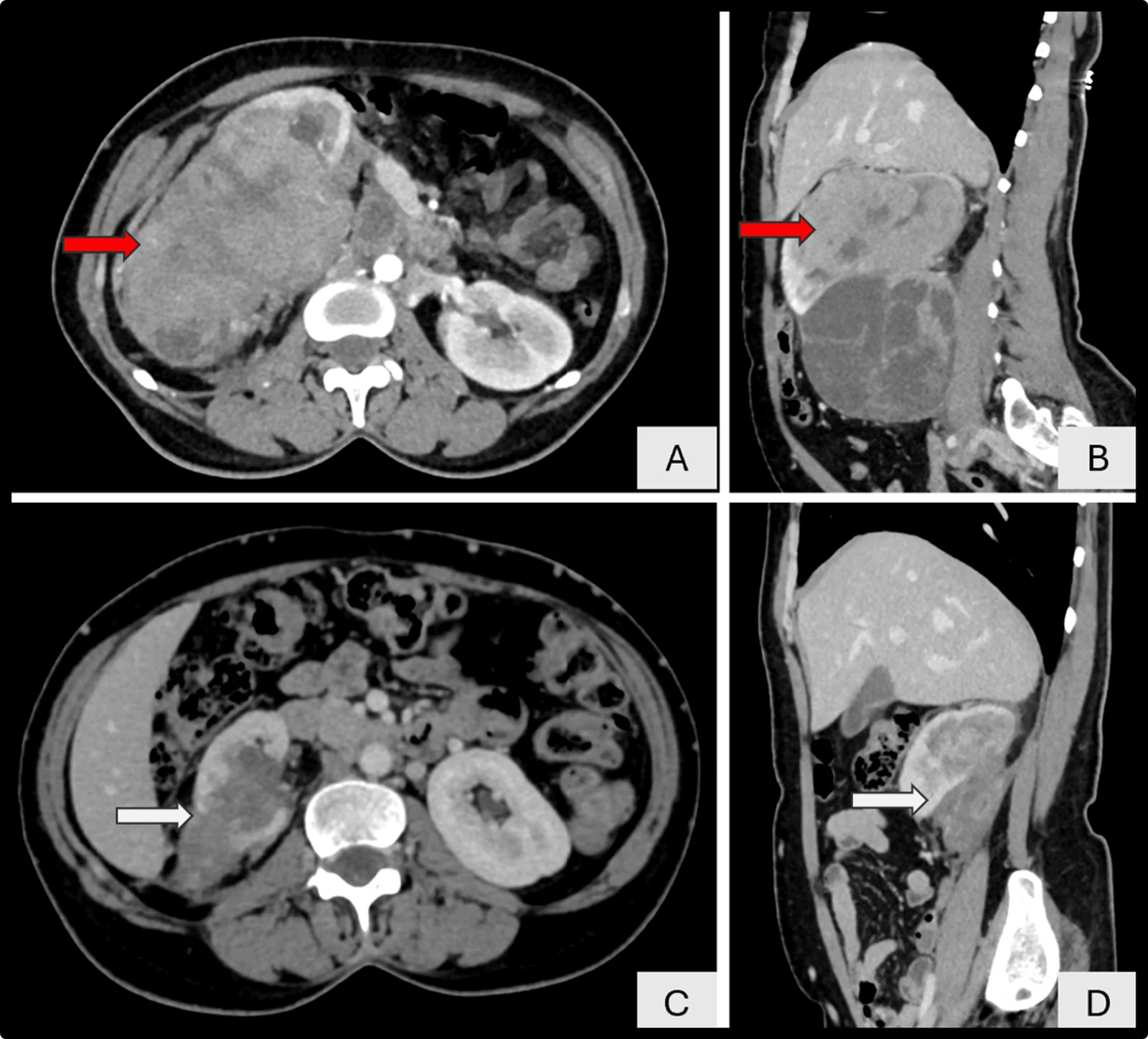
Pre (panels A and B) and post (panels C and D) chemotherapy changes A: Axial CECT image demonstrates a relatively well-defined heterogeneously enhancing right renal mass (red arrow) centered in the interpolar region, involving the corticomedullary region and adjacent calyces. The dilated IVC contains an enhancing tumor thrombus. B: Sagittal reconstructed image demonstrates the enhancing endophytic component (red arrow) and a predominantly necrotic exophytic posteroinferior component. C: Axial CECT image demonstrates a marked reduction in tumor size (white arrow) with partial resolution of the IVC tumor thrombus and reduction in IVC caliber. D: Sagittal reconstructed image demonstrates a significant reduction in the size (white arrow) of the predominantly necrotic exophytic component. CECT: Contrast-enhanced computed tomography, IVC: Inferior vena cava

The patient received systemic chemotherapy with a cyclophosphamide and adriamycin-based regimen. Follow-up CECT imaging demonstrated a marked reduction in tumor size, indicating an excellent therapeutic response (Figures 4-5).

Case two

A 63-year-old female presented with a progressively increasing abdominal lump, right flank pain, and hematuria for over seven months. There was no significant past surgical or medical history. Clinical examination revealed a large, hard, fixed mass involving the right hypochondrium, right lumbar region, and right iliac fossa. The lesion did not move with respiration and did not become prominent on leg raising, favoring a retroperitoneal origin. Mild tenderness was present without overlying skin changes.

**Figure 6:**
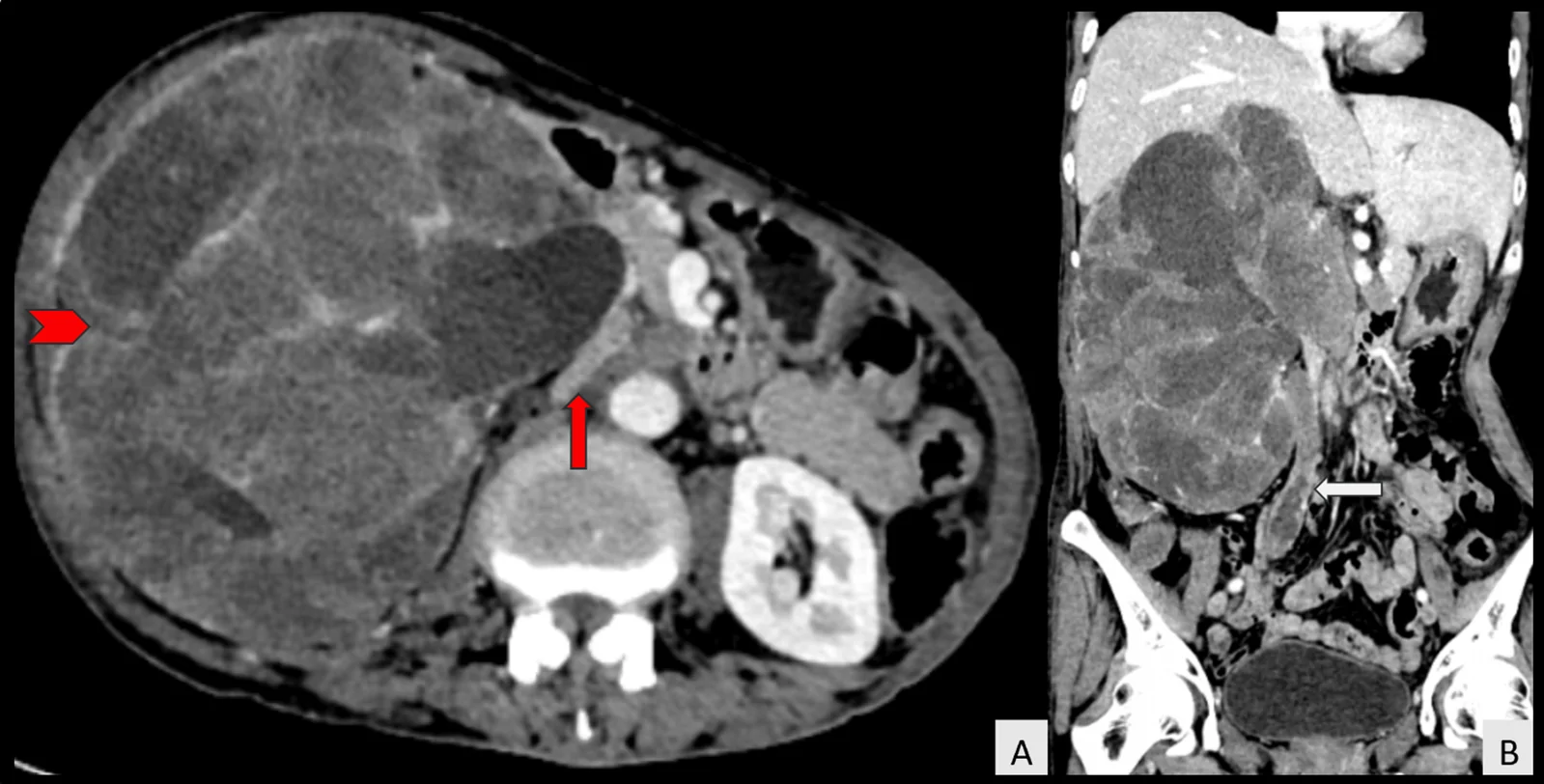
CECT of the right renal mass with collecting system involvement A: Axial corticomedullary CT urography image demonstrates a large heterogeneously enhancing right renal mass (red arrowhead) with multiple internal necrotic areas, causing marked compression of the IVC (red arrow) without evidence of vascular invasion. B: Coronal reconstructed corticomedullary phase of CT urography image demonstrates preservation of the reniform configuration of the kidney with contiguous involvement of the pelvicalyceal system and enhancing soft tissue extending along the entire right ureter up to the vesicoureteric junction (white arrow), closely mimicking transitional cell/urothelial carcinoma. IVC: Inferior vena cava

Contrast-enhanced CT demonstrated a large heterogeneously enhancing soft tissue density lesion replacing the entire right kidney, measuring approximately 16.2 × 14.3 × 19.7 cm. The native renal parenchyma was not visualized separately, confirming the renal origin of the lesion. Multiple internal necrotic areas were present. The mass demonstrated aggressive locoregional extension. Superiorly, the lesion abutted hepatic segments VI and VII with focal indistinct fat planes, raising suspicion for early hepatic infiltration. Inferiorly, the lesion extended up to the level of the L5 vertebra. Posterior extension into the paravertebral region caused significant mass effect over the right psoas muscle (Figure 6). The IVC was compressed by the lesion without infiltration. Marked displacement of bowel loops towards the left hemiabdomen was present due to large tumor bulk (Figure 6).

**Figure 7:**
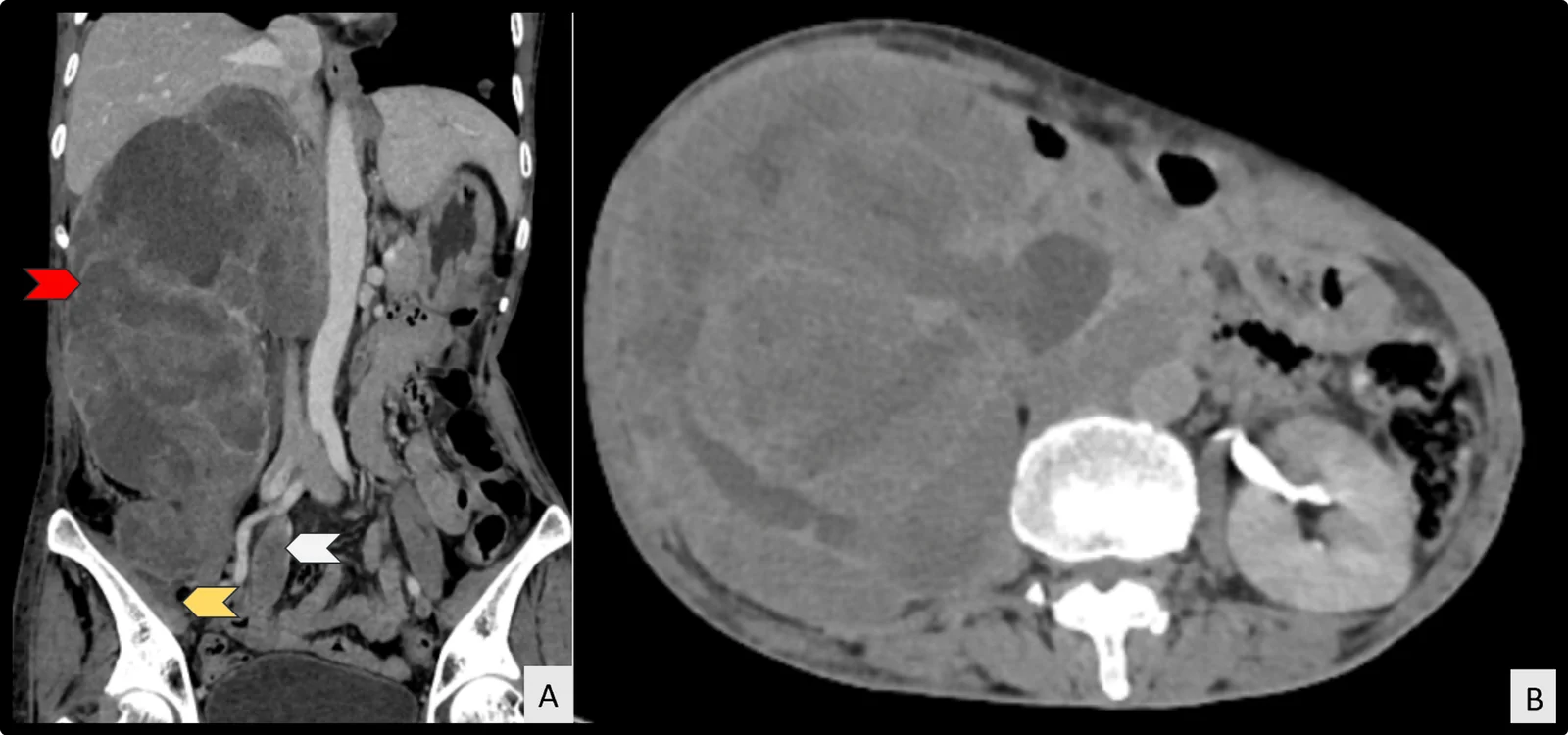
CECT of the reniform morphology and renal function A: Coronal reconstructed corticomedullary phase of the CT urography image demonstrates preservation of the reniform (bean-shaped) configuration of the enlarged right kidney despite near-complete replacement by the tumor (red arrowhead). The lesion shows extension into the lower ureter (white arrowhead) with focal infiltration into the right iliacus muscle (yellow arrowhead) B: Axial delayed excretory-phase CT urography image demonstrates absence of contrast excretion from the right kidney, indicating loss of renal function secondary to extensive tumor involvement. CECT: Contrast-enhanced computed tomography

The diffuse collecting system and ureteric involvement were unusual and diagnostically challenging features. The right ureter was dilated and demonstrated enhancing urothelial thickening with intraluminal soft tissue density extending inferiorly toward the vesico-ureteric junction, raising strong radiologic suspicion for urothelial carcinoma/transitional cell carcinoma (Figures 6-7).

Multiple heterogeneously enhancing para-aortic lymph nodes were seen consistent with nodal metastatic disease. Lung metastasis and bone metastasis were also found. The PET-CT demonstrated intense FDG avidity within the renal mass with an SUVmax of 19.45. Multiple FDG-avid para-aortic lymph nodes were identified with SUVmax reaching 13.6, consistent with nodal metastatic disease. Additional FDG-avid osseous metastatic lesions were seen involving the proximal femur, highlighting the aggressive metastatic potential of this tumor.

**Figure 8:**
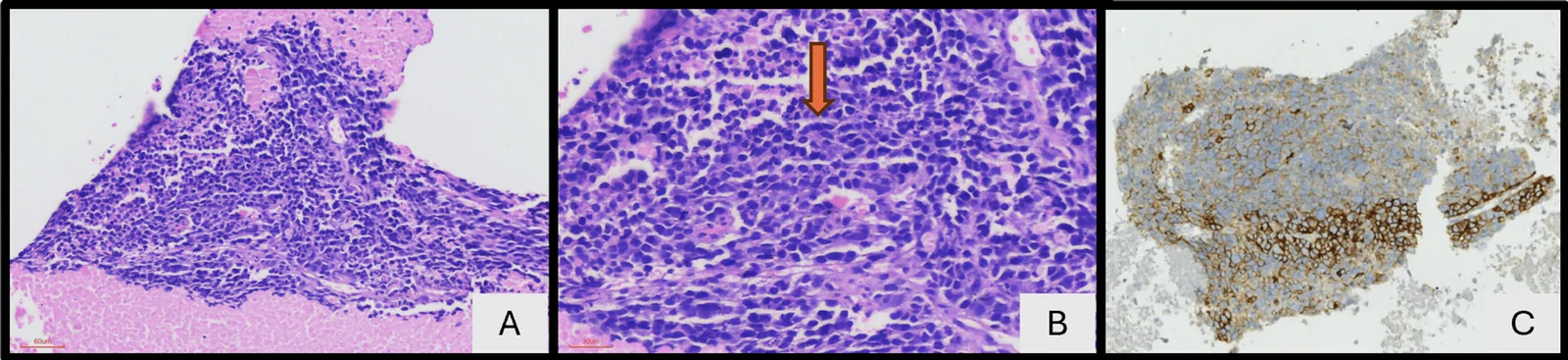
Histopathological examination and Immunohistochemical analysis reveal a malignant small round blue cell tumor A: Tumor necrosis and a small area of viable tumor (HE 10x). The tumor cells are arranged discretely, mostly scattered singly and occasional small groups. The cells are small and round with hyperchromatic nuclei. B: Tumor cells are arranged discretely, mostly scattered singly and in occasional small groups and pseudo-rosettes (orange arrow) (HE 40x). Cells are uniform-looking, small, and round with hyperchromatic nuclei and fine stippled chromatin, inconspicuous nucleoli, and scanty eosinophilic cytoplasm. C: CD99 IHC shows strong membranous positivity in tumor cells IHC: Immunohistochemistry

The imaging appearance initially favored an aggressive urothelial malignancy with extensive locoregional spread. However, given the unusually bulky infiltrative morphology, aggressive metastatic burden, and atypical imaging behavior, histopathological confirmation was pursued. Histopathological and immunohistochemical analysis of the percutaneous biopsy specimen confirmed primary renal Ewing sarcoma/PNET (Figure 8).

Both patients are currently undergoing neoadjuvant chemotherapy with ongoing clinical and imaging follow-up. The decision regarding definitive surgical management will be made following completion of neoadjuvant therapy, based on the post-treatment response and final staging.

## Discussion

Primary renal Ewing sarcoma/PNET is an exceptionally rare and highly aggressive malignancy belonging to the ESFT, accounting for less than 1% of all renal neoplasms [1-5]. The ESFT comprises a spectrum of genetically related tumors, including Ewing sarcoma of bone, extraosseous Ewing sarcoma, PNET, and Askin tumor, which share common histopathological, immunohistochemical, and molecular characteristics [2]. Although Ewing sarcoma predominantly arises from osseous structures in children and young adults, extra-skeletal involvement is uncommon, and primary renal origin represents one of the rarest manifestations [2,4,5]. Since its first description by Seemayer et al. in 1975, only a limited number of renal cases have been documented in the literature [4,7,9].

Renal Ewing sarcoma typically affects adolescents and young adults with a median age of presentation around the third decade and demonstrates a slight male predominance [1,2,4,5]. Both patients in the present series therefore represent atypical demographic presentations, particularly the second patient who presented in the elderly age group. Clinical manifestations are usually nonspecific and commonly include abdominal or flank pain, palpable abdominal mass, hematuria, constitutional symptoms, or incidental detection during imaging evaluation [1,4,5,8]. Similar nonspecific symptomatology was observed in our patients, contributing to the initial radiologic consideration of more common renal malignancies.

Radiologically, renal Ewing sarcoma remains diagnostically challenging because of significant overlap with other aggressive renal neoplasms, particularly RCC, upper tract urothelial carcinoma, Wilms tumor, lymphoma, and primary renal sarcomas [1,3-8]. Most tumors are detected at a large size and frequently demonstrate heterogeneous enhancement, extensive necrosis, hemorrhage, an infiltrative growth pattern, and aggressive locoregional extension [2,3,6]. Both tumors in our series demonstrated these characteristic aggressive imaging features.

The first case closely mimicked aggressive RCC because of extensive venous tumor thrombosis involving the renal vein and IVC. Venous invasion has been repeatedly described in renal Ewing sarcoma [1,3,4]. In our patient, the presence of an associated bland thrombus, bilateral gonadal venous collateralization, and lower limb DVT further reflected the highly aggressive vascular behavior of the tumor. Although RCC remains the most common renal tumor associated with venous tumor thrombus, certain imaging features in this case raised suspicion for an alternative diagnosis, including disproportionately large tumor size, marked necrosis, infiltrative soft tissue behavior, and absence of classic hypervascular clear cell RCC morphology.

The second case demonstrated an unusual radiologic phenotype closely simulating urothelial carcinoma/TCC. The lesion demonstrated diffuse pelvicalyceal and ureteric involvement with extension toward the vesico-ureteric junction while relatively preserving the reniform contour, thereby resembling a 'bean-type' infiltrative lesion more commonly associated with urothelial malignancy. This appearance created a significant diagnostic dilemma. Such urothelial mimicry has been infrequently emphasized in existing literature and substantially broadens the recognized imaging spectrum of renal Ewing sarcoma. In addition, the elderly age at presentation further reduced initial suspicion for this diagnosis.

Despite the nonspecific appearance, certain radiological imaging characteristics may help radiologists suspect renal Ewing sarcoma. These tumors often present as unusually large, rapidly enlarging renal masses with extensive internal necrosis and aggressive infiltrative behavior at the time of diagnosis [2,3,6]. The MRI findings frequently demonstrate marked diffusion restriction attributable to tumor hypercellularity, with significantly low apparent diffusion coefficient (ADC) values reported in prior studies [3]. Extensive vascular involvement, early metastatic disease, bulky nodal metastases, and imaging characteristics not entirely conforming to conventional RCC should also raise suspicion for a sarcomatous neoplasm [1,3,6,10].

Histopathological and immunohistochemical evaluation remains essential for definitive diagnosis because radiological imaging findings alone are underspecified [2,5,8]. Microscopically, these tumors characteristically consist of sheets of small round blue cells with scanty cytoplasm and a high nuclear-to-cytoplasmic ratio [2]. Immunohistochemically, diffuse membranous CD99 positivity is the characteristic finding, although not entirely specific [2,5]. Histopathology complemented by immunohistochemistry remains fundamental to the diagnosis, with strong membranous CD99 expression supported by additional markers including NKX2.2, FLI-1, ERG, and vimentin, which improve diagnostic accuracy, particularly in morphologically challenging cases [2,8,11]. Demonstration of EWSR1 gene rearrangement by molecular testing provides definitive confirmation of the diagnosis. Early recognition, accurate staging, and a multidisciplinary multimodal treatment approach incorporating surgery, chemotherapy, and radiotherapy are essential for optimizing clinical outcomes. To further emphasize the significant radiologic overlap and important differentiating features between renal Ewing sarcoma, RCC, and urothelial carcinoma, a comparative summary of the key imaging and pathologic characteristics is provided in Table 1.

**Table 1:** Comparative analysis between RCC, transitional cell carcinoma, and renal Ewing sarcoma/PNET RCC: Renal cell carcinoma, TCC: Transitional cell carcinoma, PNET: Primitive neuroectodermal tumor, IVC: Inferior vena cava, FDG: Fluorodeoxyglucose, IHC: Immunohistochemistry Sources: [1-5,7,8,10-13]

Feature	RCC	Urothelial carcinoma/TCC	Renal Ewing sarcoma/PNET
Typical age group	5th-7th decade	6th-8th decade	Usually adolescents and young adults; rare in elderly patients
Common clinical presentation	Hematuria, flank pain, palpable mass	Hematuria most common	Flank pain, abdominal mass, constitutional symptoms; hematuria may be absent
Tumor morphology	Usually exophytic 'ball-type' mass	Infiltrative 'bean-type' lesion preserving renal contour	Large aggressive mass; may show either ball-type or bean-type morphology
Enhancement pattern	Hypervascular enhancement especially in clear cell RCC	Mild-to-moderate delayed progressive enhancement	Heterogeneous enhancement with extensive necrosis and hemorrhage
Necrosis/hemorrhage	Common in large tumors	Less prominent	Very common and often extensive
Calcification	Occasionally present	Rare	May be present
Collecting system involvement	Secondary compression or invasion	Primary pelvicalyceal involvement typical	Can involve collecting system and mimic TCC
Ureteric extension	Uncommon	Common	Rare but may occur and mimic urothelial malignancy
Vascular Invasion	Renal vein/IVC tumor thrombus common	Less common	Relatively frequent; may show extensive venous thrombosis
Diffusion Restriction on MRI	Variable	Moderate	Often marked due to hypercellularity with low ADC values
FDG PET-CT	Variable FDG uptake	FDG avid	Usually intensely FDG avid
Metastatic pattern	Lung, bone, liver	Nodes, liver, lung	Early metastasis common; lung, bone, lymph nodes, soft tissue
Imaging clues favoring diagnosis	Hypervascular renal cortical mass with intratumoral vessels/aneurysms	Filling defects within collecting system with urothelial spread	Very large infiltrative necrotic mass, marked diffusion restriction, disproportionate vascular involvement, aggressive metastatic disease in younger patient
Histopathology	Malignant epithelial tumor	Malignant urothelial tumor	Small round blue cell tumor
Characteristic IHC markers	PAX8, RCC marker, CD10 positive	GATA3, uroplakin, CK7 positive	CD99 diffuse membranous positivity, FLI-1, NKX2.2 positive
Molecular features	VHL mutations common	FGFR3/TP53 alterations	EWSR1 translocation, commonly t(11;22)(q24;q12)
Prognostic behavior	Variable depending on subtype/stage	Aggressive when invasive	Highly aggressive with early recurrence and metastasis

## Conclusions

Primary renal Ewing sarcoma is an uncommon but highly aggressive renal malignancy with a variable radiologic spectrum that can closely mimic both renal cell carcinoma and urothelial carcinoma. Our cases demonstrate that careful analysis of subtle but atypical imaging features, including disproportionately large infiltrative masses, extensive necrosis, aggressive vascular involvement, collecting system and ureteric extension, imaging characteristics discordant with the expected phenotype of common renal malignancies, and progressive delayed enhancement and early metastatic disease, can alert the radiologist to the possibility of an alternative diagnosis. Rather than relying on a single imaging feature, recognition of this constellation of findings is critical in raising suspicion for renal Ewing sarcoma and prompting timely histopathological confirmation. This case series underscores the pivotal role of multimodality imaging not only in characterizing tumor extent but also in identifying atypical radiologic patterns that facilitate earlier diagnosis, appropriate multidisciplinary management, and assessment of treatment response.
